# Structural and Molecular Adaptations of the Placenta of Gestational Diabetes Mellitus Patients: Impact of Treatment on Vascular Endothelial Growth Factor, Vascular Endothelial Growth Factor Receptor 1, and Insulin-Like Growth Factor Expression

**DOI:** 10.7759/cureus.105445

**Published:** 2026-03-18

**Authors:** Sharmada Jois, Tejaswi H Lokanathan, Geethanjali H T, Shilpashree Y Dhananjaya

**Affiliations:** 1 Anatomy, Adichunchanagiri Institute of Medical Sciences, Adichunchanagiri University, BG Nagara, IND; 2 Anatomy, Mandya Institute of Medical Sciences, Mandya, IND; 3 Biochemistry, Farookh Academy of Medical Education, Mysuru, IND

**Keywords:** biomarkers, gestational diabetes, igf, immunohistochemistry, pregnancy, vegf

## Abstract

Background

Gestational diabetes mellitus (GDM) is a common global metabolic disorder affecting pregnant women with a rising prevalence and significant impact on the maternal-fetal outcomes. The expression of growth factors, such as vascular endothelial growth factor (VEGF) and its receptor (VEGFR1), and insulin-like growth factor (IGF), is altered in GDM, reflecting changes in placental vascularity and fetal growth regulation. Understanding these molecular alterations in GDM may help predict maternal-fetal outcomes. This study evaluates the expression of VEGF, VEGFR1, and IGF in placental tissue across different GDM treatment modalities.

Methods

This comparative cross-sectional observational study was conducted in the Department of Anatomy, Adichunchangiri Institute of Medical Sciences, BG Nagara, Mandya District, Karnataka. A total of 62 placentae were examined (31 from normoglycemic pregnancies and 31 from GDM patients). The GDM cases were further stratified into diet-controlled (n=9), oral-hypoglycemics-treated (n=11), and insulin-treated (n=11). The expression of VEGF, VEGFR1, and IGF was assessed using immunohistochemistry (IHC). Histological features, including villus morphology and vessel wall thickness, were observed with the help of hematoxylin and eosin, periodic acid-Schiff, Masson’s Trichrome, and Congo Red stains. Statistical analysis was carried out using t-tests, ANOVA, and chi-square tests.

Results

Placentae from patients with GDM, regardless of treatment modality, exhibited significantly greater positive reactivity (p<0.001) with the special stains than those of normal individuals. On IHC, IGF and VEGF expression were upregulated in placentae of insulin-treated GDM patients. IGF showed highly strong positivity in 70% of GDM cases. VEGF expression was observed in placentae of both normal and GDM cases, but the staining intensity was significantly higher in GDM placentae, especially in the syncytiotrophoblast, endothelium, and mesenchymal cells (p<0.001). VEGFR1 showed very strong positive reactivity in all GDM cases, reflecting an adaptive response to hyperglycemic conditions.

Conclusion

The structural and molecular changes observed in GDM placentae suggest that insulin treatment is associated with significant alterations in placental morphology. These findings imply that placental adaptation due to metabolic stress in GDM may vary with the intensity of treatment. The expression patterns of IGF, VEGF, and VEGFR1 may serve as potential biomarkers for monitoring placental function in relation to treatment intensity.

## Introduction

Gestational diabetes mellitus (GDM) is a complex metabolic disorder that alters the intrauterine environment, primarily affecting placental development, structure, and function [1]. The placenta is an important link between the mother and the developing fetus and undergoes substantial molecular and cellular adaptations in the hyperglycemic condition characteristic of GDM [2]. The maternal hyperglycemia causes increased glucose transport in the placenta, leading to fetal hyperinsulinemia and macrosomia. Hyperglycemia induces oxidative stress and inflammatory changes, leading to altered placental morphology and vascular architecture [3,4]. These adaptations may result from changes in the expression of angiogenic and growth factors, which are essential for placental growth and vascularization.

Development and angiogenesis in the placenta are complex processes regulated by numerous growth factors. The angiogenic condition in GDM may involve dysregulated expression of key regulatory factors, such as vascular endothelial growth factor (VEGF), its receptor (VEGFR1), and insulin-like growth factor (IGF) [5]. These factors play a very important role in placental vascular development, trophoblast function, and nutrient transport capacity; altered expression of these factors may affect the maternal and fetal outcomes [6,7].

The proliferation and migration of endothelial cells and the formation of new blood vessels (angiogenesis) are mainly regulated by VEGF and VEGFR1 [8]. VEGF promotes endothelial cell proliferation, migration, and vascular permeability, while VEGFR1 acts as a decoy receptor and controls the VEGF by binding to it and modulating the bioavailability of VEGF [9]. Altered expression of VEGF and VEGFR1 in GDM can lead to hypervascularization, which can disrupt the placental vascular architecture [6].

IGF plays a vital role in placental growth, development and nutrient transport [10,11]. IGF has a significant contribution in proliferation, differentiation, and invasion of the trophoblast; the bioavailability of IGF depends on IGF binding protein [12]. The altered expression of IGF in GDM pregnancies increases nutrient supply, leading to increased placental weight and fetal overgrowth [13].

Currently, GDM management includes dietary modification, oral hypoglycemic agents (metformin), and insulin therapy. Each type of treatment can affect maternal glycemic control. This, in turn, influences overall placental modifications [14]. Optimizing maternal and fetal outcomes is important, yet the comprehensive and differential impact of these treatments on placental morphology, vasculature, and angiogenic factor expression remains poorly understood. Understanding treatment-specific changes in VEGF, VEGFR1, and IGF expression is very important. It provides insight into the molecular mechanisms underlying placental modifications to metabolic stress. This knowledge may help develop targeted therapies and biomarker strategies to manage GDM patients [7]. The present study aimed to compare morphometric features, histopathological changes, and immunohistochemical expression of VEGF, VEGFR1, and IGF in placentae from GDM and normal-pregnancy groups. 

## Materials and methods

Study design and participants

This comparative cross-sectional observational study was conducted in the Department of Anatomy, Adichunchangiri Institute of Medical Sciences, BG Nagara, Mandya District, Karnataka. The study protocol was approved by the Institutional Ethics Committee, and written informed consent was obtained from the study participants. A total of 62 placental specimens were analyzed, including 31 from normal pregnancies (controls) and 31 from pregnancies complicated with GDM. All GDM cases were diagnosed and confirmed by an oral glucose tolerance test according to the Diabetes in Pregnancy Study Group of India (DIPSI) [15]. Placentae from pregnant women diagnosed with GDM using DIPSI criteria, singleton pregnancies, term deliveries (≥37 weeks), and placentae obtained immediately after delivery were included in the study. Placentae from pregnancies complicated by pre-existing diabetes mellitus, hypertensive disorders of pregnancy, multiple gestations, congenital fetal anomalies, maternal infections, and placental abruption or placenta previa were excluded from the study. 

Placental collection and tissue processing

Placental samples were collected immediately after delivery. Membranes were trimmed, and the umbilical cord was trimmed 2 cm from the site of insertion. The following placental morphometric parameters were evaluated: placental weight (in grams) was determined using a calibrated electronic weighing scale; the number of cotyledons was counted on the maternal surface; the placental diameter was measured at two distinct points with a measuring tape, and the mean value was recorded. The central placental thickness was measured at the center using Vernier calipers, while the peripheral thickness was measured at four equidistant points along the placental margins and averaged.

Two to three tissue samples were obtained from the central, intermediate, and peripheral zones of each placenta. Samples were fixed and stored in 10% neutral buffered formalin for 24 hours. Tissues were then dehydrated through graded alcohols, then cleared with Xylene and embedded in paraffin wax. Serial sections of 4-5 μm thickness were prepared for histopathological and immunohistochemical analysis. 

Histopathological examination

Sections were stained with hematoxylin and eosin (H & E) for routine histopathological evaluation. All slides were examined under light microscopy. Histomorphometric measurements were conducted using a Cilika BT-E (2021) digital benchtop microscope (MedPrime Technologies Pvt. Ltd., Thane, India) with an integrated tablet display at 10× and 40× magnifications. For each slide, 10 randomly selected microscopic fields were examined, and mean values were calculated. Quantitative parameters included villi count per field (grid method), villous diameter and perimeter (mm, at 40×), syncytiotrophoblast thickness (mm), capillary count per villus, capillary diameter (mm), and thickness of stem villi blood vessels (mm). Qualitative parameters assessed were the presence of syncytial knots (categorized as less or more), nucleated red blood cells (NRBCs) in fetal capillaries, villous degeneration, immature villi, intervillous space (wide or narrow), villous edema, avascular villi, villous fibrinoid degeneration, chorangiosis (≥10 capillaries in ≥10 terminal villi in ≥10 fields), vascular congestion, capillary edema, thrombosis, and calcification. Each qualitative parameter was recorded as present or absent unless otherwise specified.

Immunohistochemical analysis

Immunohistochemical staining was performed on all 62 placental tissue samples using the following primary antibodies: anti-IGF-1 antibody (polyclonal; dilution 1:100; BioCare Medical), anti-VEGF antibody (monoclonal; dilution 1:50; Elabscience, USA), and anti-VEGFR1/Flt-1 antibody (polyclonal; dilution 1:50; Elabscience, USA)

The staining procedure included the following steps: deparaffinization in xylene and rehydration through graded alcohols; heat-induced antigen retrieval with EDTA buffer; incubation with primary antibodies; detection with a horseradish peroxidase-based system using diaminobenzidine as the chromogen; and counterstaining with hematoxylin. Positive control placental tissue with known high expression was included in each staining batch. Negative controls were prepared by omitting the primary antibody.

IGF-1 expression was assessed in syncytiotrophoblast, cytotrophoblast, apical cytoplasm, mesenchymal connective tissue, and fetal blood vessels. VEGF and VEGFR1 expression were evaluated in syncytiotrophoblast, cytotrophoblast, endothelial cells, capillary endothelium, vascular endothelium, vascular smooth muscle cells, and mesenchymal cells.

Immunohistochemical expression intensity was assessed using a standardized German semi-quantitative scoring system: Score 0: No staining (negative expression); Score 1: Weak staining (light brown); Score 2: Moderate staining (medium brown); and Score 3: Strong staining (dark brown) [16]. All sections were independently evaluated by the first two authors using predefined criteria, who were blinded to the clinical grouping (normal or GDM) of the specimens. The observations were cross-validated by an experienced pathologist who was also blinded to the study group allocation. The final scores were recorded for statistical analysis.

Statistical analysis

Statistical analysis was performed using IBM SPSS Statistics for Windows, Version 20 (Released 2011; IBM Corp., Armonk, New York, United States). Continuous variables were expressed as mean ± standard deviation and compared using the independent samples t-test (two groups) or one-way ANOVA (multiple groups), as appropriate. Categorical variables were analyzed using the chi-square test. A two-tailed p-value < 0.05 was considered statistically significant.

## Results

Morphometric comparison

The number of cotyledons in GDM placentae (20.87 ± 3.26) was significantly higher (p<0.001) than that in the normal placentae (13.06 ± 3.00). Other morphometric parameters did not show any significant trends.

The diet-controlled GDM placentae (2392.22 ± 402.82 g) showed significantly reduced birth weights compared to normal placentae (2816.13 ± 302.78 g, p=0.002). The insulin-treated pregnancies demonstrated significantly increased placental weight (750.22 ± 228.56 g versus normal 632.51 ± 125.41 g, p=0.046), indicating placental hypertrophy. The feto-placental ratio was significantly reduced (3.80 ± 0.90 versus normal 4.55 ± 0.54, p=0.003), suggesting disproportionate placental enlargement compared to fetal size (Table 1).

**Table 1:** Treatment Modality-Specific Comparison of Morphometric Data *Statistically significant (p<0.05). p-values calculated using an independent samples t-test. t-values represent the test statistic for each comparison. Data presented as mean ± standard deviation. GDM: Gestational diabetes mellitus

Parameter	Normal (n=31)	GDM Diet-Controlled (n=9)	t-Value	p-Value (vs Normal)	GDM Oral Hypoglycemic-Treated (n=11)	t-Value	p-Value (vs Normal)	GDM Insulin-Treated (n=11)	t-Value	p-Value (vs Normal)
Placental Weight (grams)	632.51 ± 125.41	624.83 ± 150.55	t=-0.14	0.88	653.94 ± 114.03	t=-0.49	0.63	750.22 ± 228.56	t=2.05	0.046*
Number of Cotyledons	13.06 ± 3.00	20.89 ± 2.73	t=-7.02	<0.001*	18.64 ± 2.42	t=-6.24	<0.001*	23.09 ± 2.84	t=-10.35	<0.001*
Central Thickness (cm)	2.06 ± 0.52	1.73 ± 0.35	t=1.71	0.09	2.09 ± 0.31	t=-0.19	0.85	1.98 ± 0.49	t=0.44	0.66
Peripheral Thickness (cm)	1.14 ± 0.37	1.13 ± 0.34	t=0.01	0.99	1.10 ± 0.20	t=0.29	0.77	1.12 ± 0.28	t=0.14	0.89
Placental Diameter (cm)	17.65 ± 2.12	17.33 ± 0.94	t=0.43	0.67	17.14 ± 2.04	t=0.68	0.5	17.95 ± 2.11	t=-0.39	0.7
Birth Weight (grams)	2816.13 ± 302.78	2392.22 ± 402.82	t=3.30	0.002*	2842.73 ± 351.91	t=-0.23	0.82	2671.82 ± 508.34	t=1.11	0.27
Feto-Placental Ratio	4.55 ± 0.54	4.12 ± 1.46	t=1.32	0.19	4.45 ± 0.87	t=0.42	0.67	3.80 ± 0.90	t=3.15	0.003*

Histopathological comparison

**Figure 1:**
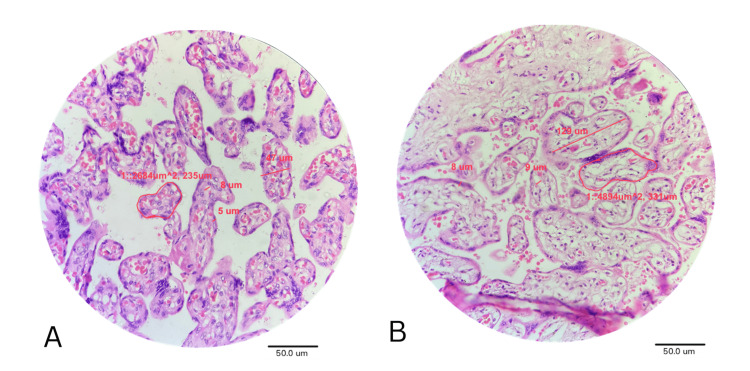
H and E-Stained Images of Normal Placenta (A) and GDM Placenta (B) under 40x Magnification A: Normal placenta showing regular villous architecture with minimal syncytial mass and well-defined intervillous space; B: GDM placenta showing villous hypertrophy, increased syncytial mass, capillary congestion, and decreased intervillous space. H: Hematoxylin; E: Eosin; GDM: Gestational diabetes mellitus.

GDM groups demonstrated a significant increase in all continuous parameters. Villous hypertrophy, villous perimeter, and trophoblast thickness are significantly greater in oral hypoglycemic-treated placentas compared to those treated with other modalities. Capillary diameter and blood vessel wall thickness were more significantly pronounced in the insulin-treated group (Figure 1, Table 2).

**Table 2:** Treatment Modality-Specific Comparison of Continuous Histopathological Data ***p<0.001 (highly significant; indicates extremely strong statistical evidence of difference between groups). p-values calculated using an independent samples t-test. t-values represent the test statistic for each comparison; negative t-values indicate that the gestational diabetes mellitus (GDM) group has a higher mean than the normal group. Data presented as mean ± standard deviation. mm: millimeters

Parameter	Normal (n=31)	GDM Diet-Controlled (n=9)	t-Value	p-Value	GDM Oral Hypoglycemic-Treated (n=11)	t-Value	p-Value	GDM Insulin-Treated (n=11)	t-Value	p-Value
Number of Villi	119.03 ± 1.40	162.11 ± 7.55	t=-17.45	<0.001***	146.73 ± 9.55	t=-13.82	<0.001***	149.00 ± 19.90	t=-7.18	<0.001***
Villous Diameter (mm)	0.0569 ± 0.01	0.0730 ± 0.01	t=-4.23	<0.001***	0.0805 ± 0.002	t=-8.92	<0.001***	0.0795 ± 0.003	t=-7.86	<0.001***
Villous Perimeter (mm)	0.2542 ± 0.02	0.3080 ± 0.06	t=-3.68	<0.001***	0.3345 ± 0.05	t=-6.45	<0.001***	0.3111 ± 0.05	t=-4.89	<0.001***
Trophoblast Thickness (mm)	0.003 ± 0.001	0.0075 ± 0.001	t=-12.56	<0.001***	0.0086 ± 0.001	t=-15.34	<0.001***	0.0075 ± 0.002	t=-9.82	<0.001***
Capillary Diameter (mm)	0.0156 ± 0.003	0.0183 ± 0.005	t=-5.67	<0.001***	0.0223 ± 0.008	t=-6.89	<0.001***	0.0235 ± 0.003	t=-8.92	<0.001***
Blood Vessel Wall Thickness (mm)	0.0153 ± 0.004	0.0264 ± 0.003	t=-8.45	<0.001***	0.029 ± 0.009	t=-9.23	<0.001***	0.0396 ± 0.009	t=-12.67	<0.001***

All categorical parameters were significantly increased in the GDM groups. Chorangiosis was observed in all treatment modalities for GDM. Diet-controlled GDM showed the highest percentage of nucleated RBCs and calcification, suggesting that diet control, despite the lowest-intensity treatment, shows the highest signs of fetal hypoxia, premature ageing, and placental degeneration compared with other treatment modalities.

Villous degeneration was observed in 63-100% of placentae. Insulin-treated patients presented increased percentage of villous edema, capillary edema, blood vessel congestion and thrombosis, suggesting that placental pathology progresses with treatment intensity, leading to severe vascular dysfunction (Table 3).

**Table 3:** Treatment Modality-Specific Comparison of Categorical Histopathological Data ***p<0.001 (highly significant; indicates extremely strong statistical evidence of difference between groups). Data presented as number of cases/total (percentage).  Statistical significance was assessed using the chi-square test. All categorical variables showed p<0.001 when comparing gestational diabetes mellitus (GDM) groups (combined) versus the normal group. RBCs: Red blood cells; GDM: Gestational diabetes mellitus.

Histopathological Finding	Normal n/N (%)	Diet-Controlled n/N (%)	Oral Hypoglycemic n/N (%)	Insulin-Treated n/N (%)	All GDM n/N (%)	χ²-Value for All GDM vs Normal	p-Value
Nucleated RBCs Present	0/31 (0.0%)	4/9 (44.4%)	2/11 (18.2%)	2/11 (18.2%)	8/31 (25.8%)	7.03	0.008^**^
Villous Degeneration	0/31 (0.0%)	9/9 (100.0%)	7/11 (63.6%)	11/11 (100.0%)	27/31 (87.1%)	44.35	<0.001^***^
Immature Villi	0/31 (0.0%)	6/9 (66.7%)	3/11 (27.3%)	6/11 (54.5%)	15/31 (48.4%)	17.24	<0.001^***^
Villous Edema	0/31 (0.0%)	6/9 (66.7%)	7/11 (63.6%)	9/11 (81.8%)	22/31 (71.0%)	31.07	<0.001^***^
Avascular Villi	0/31 (0.0%)	5/9 (55.6%)	3/11 (27.3%)	6/11 (54.5%)	14/31 (45.2%)	15.59	<0.001^***^
Chorangiosis	0/31 (0.0%)	9/9 (100.0%)	11/11 (100.0%)	11/11 (100.0%)	31/31 (100.0%)	58.06	<0.001^***^
Blood Vessel Congestion	0/31 (0.0%)	7/9 (77.8%)	9/11 (81.8%)	11/11 (100.0%)	27/31 (87.1%)	44.35	<0.001^***^
Capillary Edema	0/31 (0.0%)	3/9 (33.3%)	6/11 (54.5%)	11/11 (100.0%)	20/31 (64.5%)	26.65	<0.001^***^
Thrombosis	0/31 (0.0%)	0/9 (0.0%)	5/11 (45.5%)	11/11 (100.0%)	16/31 (51.6%)	18.95	<0.001^***^
Placental Calcification	0/31 (0.0%)	9/9 (100.0%)	5/11 (45.5%)	8/11 (72.7%)	22/31 (71.0%)	31.07	<0.001^***^

Immunohistochemistry comparison

IGF Expression Pattern

**Figure 2:**
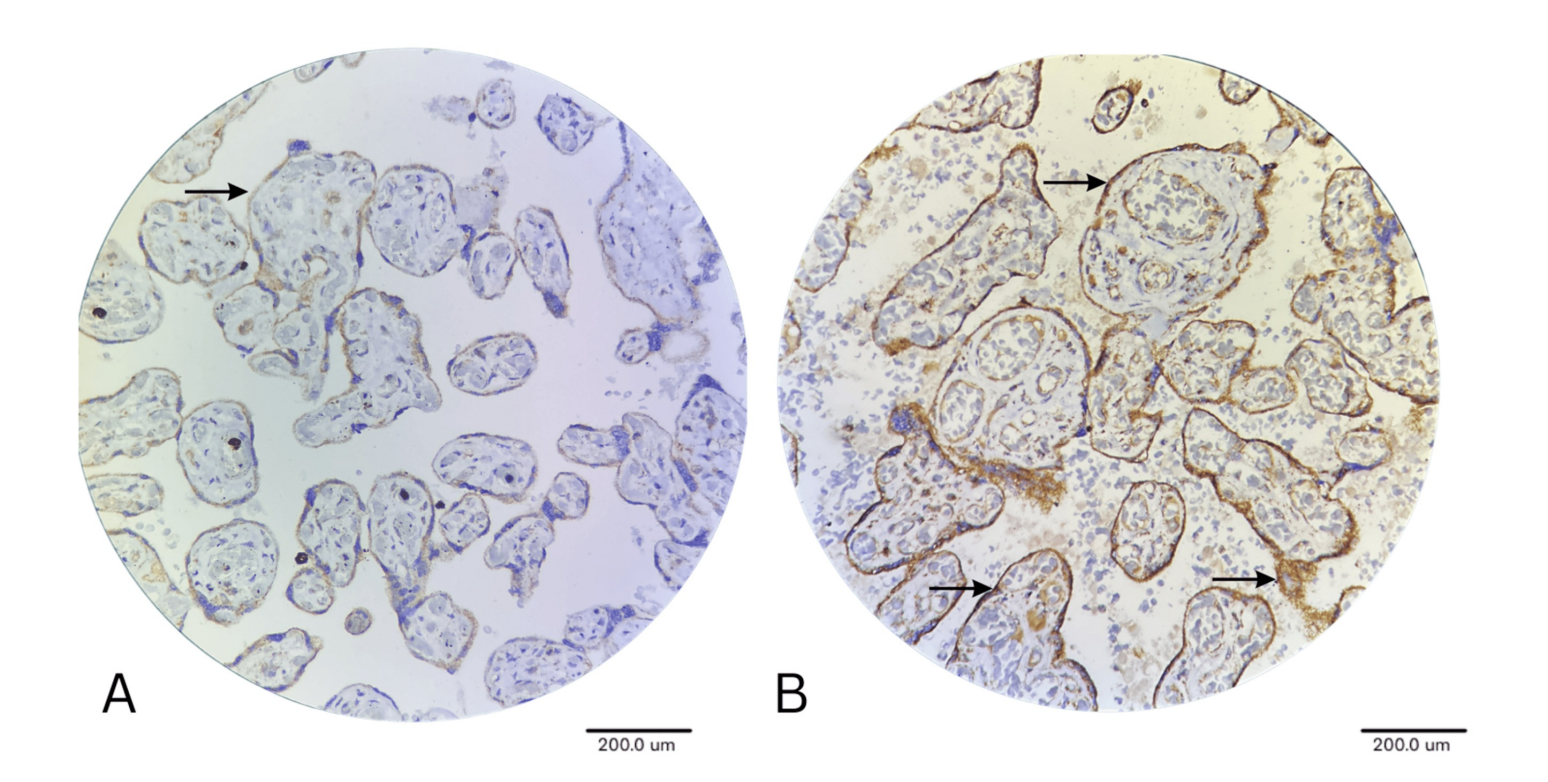
IHC Image Showing IGF Expression in Normal (A) and GDM Placenta (B) under 40x Magnification A: Normal placenta, arrow showing weak to negative IGF expression in cytotrophoblast and syncytiotrophoblast; B: GDM placenta, arrows showing strong positive (dark brown) IGF expression in cytotrophoblast and syncytiotrophoblast, indicating IGF upregulation GDM: Gestational diabetes mellitus; IGF: Insulin-like growth factor; IHC: Immunohistochemistry

IGF expression analysis revealed a drastic alteration in the GDM placentae compared to the normal placentae. A strong staining pattern was observed in syncytiotrophoblast (2.62± 0.66, p<0.001) and cytotrophoblast (2.50± 0.92, p<0.03), suggesting significant upregulation in GDM compared with the normal placenta, where there is none. This increased pattern indicates a dramatic metabolic adaptation in the primary hormone-producing cell layer of the placenta. Maternal and fetal parts of the placenta also exhibited increased gradients in IGF expression according to treatment modalities, with less in diet-controlled, moderate in tablet therapy and highest in insulin-treated cases (Figure 2).

Treatment-Specific IGF Expression

Diet-controlled GDM exhibited maximum IGF expression in syncytiotrophoblast and cytotrophoblast (mean score: 2.6). This suggests that mild hyperglycemia induces IGF upregulation. Oral hypoglycemic-related cases demonstrated intermediate IGF expression in the trophoblast (mean score: 2.4). Insulin-treated cases exhibited the highest IGF expression in syncytiotrophoblast and cytotrophoblast (mean score: 2.6), suggesting that the best treatment can also cause severe metabolic disruption.

VEGF Expression Pattern

**Figure 3:**
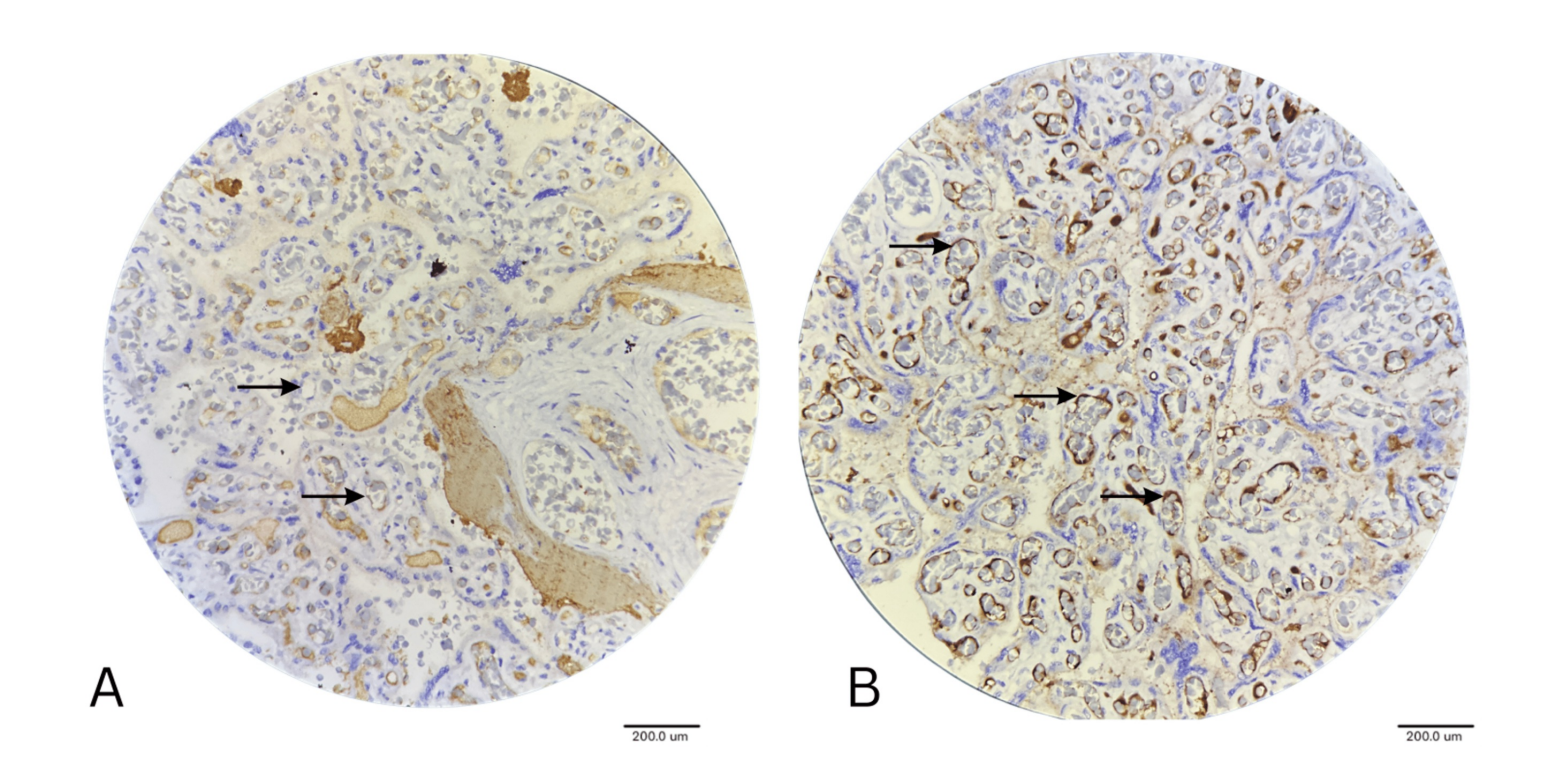
IHC Image Showing Normal Placenta (A) VEGF Expression in GDM Placenta (B) under 40x Magnification Arrows pointing to non-expression of VEGF in the blood vessel of normal placenta in (A), a strongly positive expression of VEGF on the blood vessel endothelium of GDM placenta in (B). VEGF: Vascular endothelial growth factor; GDM: Gestational diabetes mellitus

The VEGF expression pattern was significantly altered in GDM cases compared to normal cases. VEGF expression was uniformly absent in the syncytiotrophoblast of both normal and GDM cases, but VEGF showed slightly elevated expression in GDM (0.09±0.30) cases compared to normal (0.00) (Figure 3). Treatment-specific VEGF expression in vascular tissues: Endothelial cell VEGF expression showed highly significant variation (F23.889, p<0.001), with insulin-treated placentas demonstrating moderate to strong expression (score 1.91± 0.70), compared to diet control (score 1.00) and oral hypoglycemic therapy (score 0.42± 0.51) (Figure 3). In capillary endothelium, VEGF showed progressively higher expression across the treatment modalities (F=9.087, p=0.001). No expression in normal (0.00), diet-controlled (0.11±0.33), oral hypoglycemic-treated (0.42±0.5), and insulin-treated (1.27±0.90). This suggests treatment intensity-specific increased capillary angiogenic activation. VEGF demonstrated significant differences in its expression in mesenchymal cells (F25.375, p<0.001), with insulin-treated placentae showing markedly elevated expression (score 2.45± 0.93) compared to uniform moderate expression in diet control and tablet therapy groups (both score 1.0).

VEGFR1 Expression Pattern

**Figure 4:**
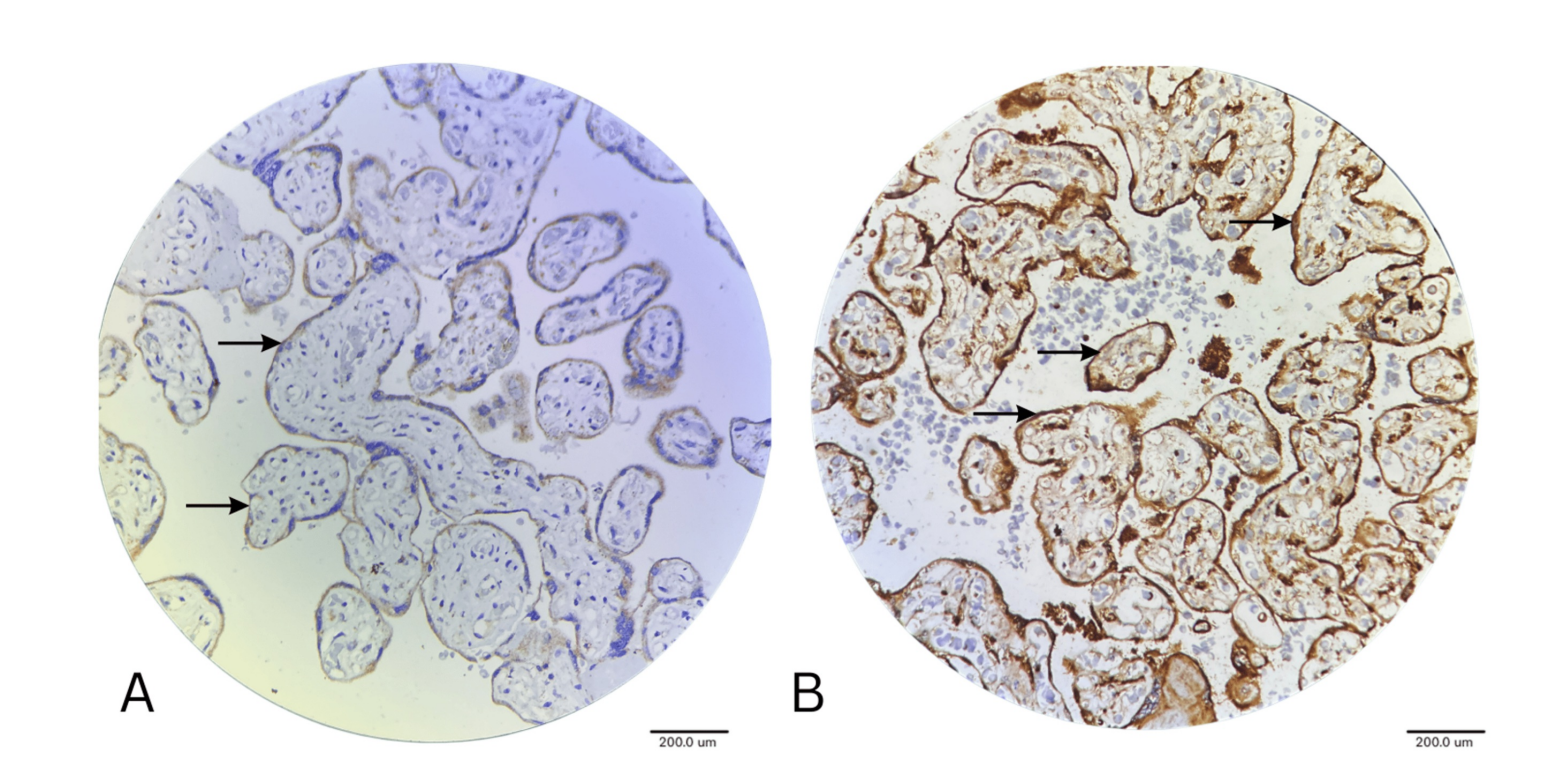
IHC Image Showing VEGFR1 Expression in Normal Placenta (A) and GDM Placenta (B) under 40x Magnification Arrows pointing to a positive expression of VEGFR1 on the cytotrophoblast and syncytiotrophoblast in the normal placenta in (A), arrows pointing to a strongly positive expression of VEGFR1 on the cytotrophoblast and syncytiotrophoblast in the GDM placenta in (B). IHC: Immunohistochemistry

VEGFR1 expression analysis revealed the most consistent and significant altered expression across all GDM placentae compared to normal placentae. This consistent upregulation reflects the adaptive mechanism of the placenta in diabetic pregnancies. Syncytiotrophoblast and cytotrophoblast showed a massive upregulation, nearly threefold increase compared to the normal placenta (2.88±0.49 in GDM, 1.00±0.00 in normal, p<0.001). This indicated that the coordinated receptor expression changes across the trophoblast in response to metabolic stress (Tables 4, 5; Figure 4).

**Table 4:** Summary of Treatment Modality-Specific IGF, VEGF, and VEGFR1 Expression IGF: Insulin-like growth factor; VEGF: Vascular endothelial growth factor; VEGFR1: Vascular endothelial growth factor receptor 1; GDM: Gestational diabetes mellitus.

Finding	Normal	GDM (Diet-Controlled Group)	GDM (Oral Hypoglycemic-Treated Group)	GDM (Insulin-Treated Group)
IGF expression summary				
Syncytiotrophoblast	Negative/Weak	Very strong positive	Weak to moderate positive	Strong positive
Cytotrophoblast	Weak positive	Strong positive	Weak to moderate positive	Strong positive
Mesenchymal tissue	Negative	Strong positive	Negative	Strong positive, with pale fetal part
VEGF expression summary				
Endotheliocyte	Negative	Weak positive	Negative	Moderate to strong positive
Capillary endothelium	Negative	Weak positive	Negative	Positive to strong positive
Mesenchymal tissue	Weak positive	Positive	Positive	Strong positive
VEGFR1 expression summary				
Syncytiotrophoblast	Positive	Strong positive	Strong positive	Strong positive
Cytotrophoblast	Positive	Strong positive	Strong positive	Strong positive
Mesenchymal tissue	Weak positive	Strong positive	Strong positive	Strong positive

**Table 5:** Treatment Modality-Specific IGF, VEGF, and VEGFR1 Expression Scoring Chart Strong Positive: number with strong positive expression; %: percentage with strong positive expression; Mean: mean scoring value. N (n): sample size; IGF: Insulin-like growth factor; VEGF: Vascular endothelial growth factor; VEGFR1: Vascular endothelial growth factor receptor 1; GDM: Gestational diabetes mellitus.

Treatment Group	N	VEGFR1 Strong Positive (n)	VEGFR1 Strong Positive (%)	VEGFR1 Mean Score	VEGF Strong Positive (n)	VEGF Strong Positive (%)	VEGF Mean Score	IGF Strong Positive (n)	IGF Strong Positive (%)	IGF Mean Score
	(Sample Size)									
Normal	31	0	0.00%	1	0	0.00%	0	0	0.00%	0
GDM Diet-Controlled	9	9	100.00%	3	0	0.00%	0.11	8	88.90%	2.6
GDM Oral Hypoglycemic-Treated	11	11	100.00%	2.91	4	36.40%	0.75	10	90.90%	2.4
GDM Insulin-Treated	11	10	90.90%	2.91	8	72.70%	1.82	10	90.90%	2.6
All GDM Combined	31	30	96.80%	2.94	12	38.70%	0.89	28	90.30%	2.53

Treatment-Specific VEGFR1 Expression in Vascular Tissues

**Figure 5:**
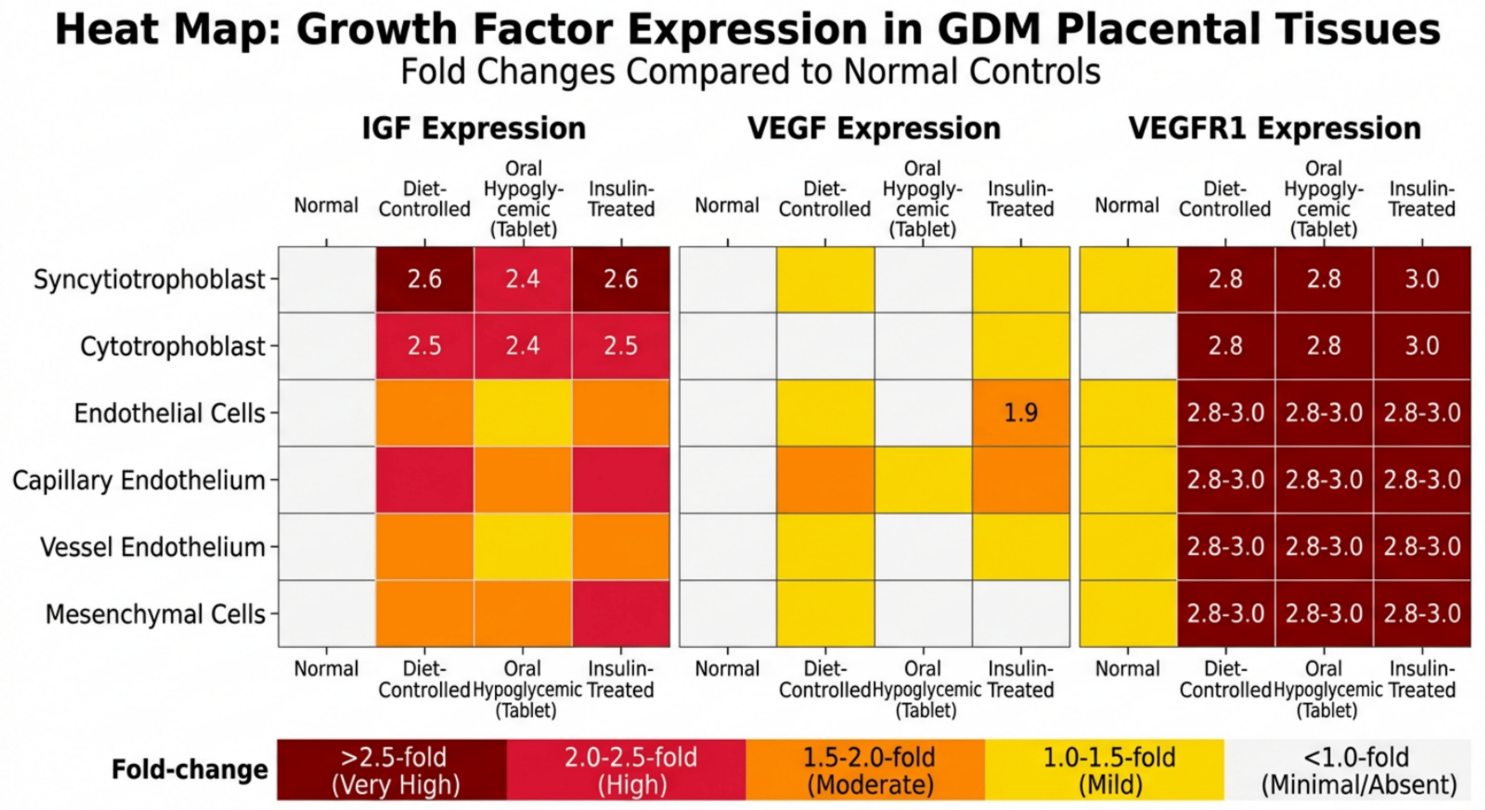
Heat Map of Growth Factor Expression in GDM and Normal Placentae IGF: Insulin-like growth factor; VEGF: Vascular endothelial growth factor; VEGFR1: Vascular endothelial growth factor receptor 1; GDM: Gestational diabetes mellitus

In the vessel endothelium, there was an increased VEGFR1 expression from 1.00±0.00 in normal to 2.81±0.59 in GDM cases (p<0.001). Insulin-treated cases showed the highest endothelial expression (2.64±0.81), thus indicating enhanced angiogenic signaling in placental cells for VEGF action. Endothelial gradient was 1.00. in diet-controlled cases, 0.42 in oral hypoglycemic-treated patients and 1.91 in insulin-treated patients. Smooth muscle of the vessels showed significantly increased expression levels of VEGFR1 in GDM (1.75±0.98, p=0.019). This represents the DE novo receptor expression leading to altered vascular wall signaling mechanisms. Mesenchymal cells showed threefold upregulation of VEGFR1, indicating overall mesenchymal activation in the GDM placenta (2.45, p<0.001) compared to the normal placenta (1.00) (Figure 5).

## Discussion

Morphometric adaptations in GDM

The findings of the present study reveal that GDM induces significant placental morphometric changes, such as increased placental weight and maternal cotyledon count (60% increases; p<0.001) compared with normal cases. These findings support the previous observations [17,18]. This represents compensatory hyperplasia in response to maternal hyperglycemia. This adaptation is required to maintain the nutrient transfer despite an altered metabolic environment. 

Disproportionate increased placental weight compared to fetal weight reflects a reduced feto-placental ratio, which indicates that there is an insufficient placental function. These findings are also reported by Daskalkis et al. [3]. Hyperplasia in the GDM placenta appears to be a protective mechanism to maintain proper foetal nutrition supply, though it may not normalize the placental insufficiency [19,20].

Histopathological adaptations in GDM

Villous hypertrophy, degeneration, and immaturity were the most consistent histological findings in this study. Villous immaturity, along with trophoblastic layer thickening, reduced villous size, increased Hofbauer cell numbers, and chorangiosis, directly impairs placental efficiency. Similar histopathological changes affecting fetal development have been reported in patients with GDM [21,22]. Increased syncytial knots, frequently observed in GDM, were also noted in this analysis. This study further identified prominent cytotrophoblastic hyperplasia. These changes impair oxygen and nutrient diffusion, leading to placental hypoxia and compensatory angiogenic responses [23,24]. 

Another significant finding from our study was the presence of villous edema and capillary edema. These findings have been reported in previous studies. In the present study, insulin-treated cases demonstrated a significant increase, suggesting a relationship between fluid homeostasis and insulin signaling [25]. Increased placenta blood flow causes increased hydrostatic pressure in the fetal capillary, which may lead to the villous and capillary oedema in the GDM placenta [26,27].

Growth factor expression pattern

The present study reveals that GDM induces enormous alterations in placenta angiogenic factor expression. This study quantifies the magnitude and consistency of treatment-specific effects on these factors, which cause dramatic dysregulation in the placental molecular environment. The present study provides new insight into the pathophysiology of diabetic placental disruption. 

IGF system alterations

Studies done on animals suggest that fetal growth is influenced by maternal IGF [28]. The study by Hiden et al. reported dysregulation of IGF and its receptors in diabetic pregnancies, findings that correlate with our study, which reports dramatic upregulation of IGF in GDM trophoblasts [29]. A study by Abu-Amero et al. suggests increased IGF and its receptors in IUGR births compared with normal births, findings that correlate with our study and likely reflect an adaptive mechanism [30].

A treatment-specific pattern of IGF expression provides important insights into metabolic control mechanisms. The contradictory finding of the highest increase in IGF expression in diet-controlled cases suggests that mild hyperglycemic conditions elicit the greatest compensatory responses compared with other treatment modalities [2].

VEGF & VEGFR1 system alteration

Pietro et al. found strong positive VEGF expression in trophoblastic cells of GDM placentas, which directly contradicts our study, in which it was absent or very weak. VEGFR1 expression in both studies shows significant upregulation in the GDM placenta. Both studies exhibit VEGFR1 expression in vascular and trophoblastic cells [1].

Meng et al. reported a universal decrease in VEGF expression, a finding also observed in the present study. In trophoblastic tissue, VEGF expression was minimal or absent, consistent with previous research. In contrast, VEGFR1 demonstrated compartment-specific increases in expression within vascular and mesenchymal tissues [31].

Our study also showed results similar to those of Bhattacharjee et al., who found minimal VEGF expression in the endothelium of overt diabetic cases [8].

Troncoso et al. reported decreased VEGFR1 mRNA and protein levels in GDM, whereas our study observed a significant increase in expression across almost all placental tissue compartments [6]. This study was tissue-specific, as it used PCR; in our study, we observed expression at the cellular level.

VEGF expression patterns in our study reveal subtler yet significant treatment-dependent alterations. While the overall comparison with the normal group didn’t show statistical significance, there was a progressive increase in endothelial and mesenchymal expression across treatment modalities. This increased expression gradient suggests an angiogenic drive that correlates with metabolic stress. 

The VEGFR1 expression pattern in our study reveals the most striking finding: significant upregulation across all placental tissue compartments in GDM cases. These changes may contribute to altered vascular reactivity and remodeling observed in GDM cases. This also indicates a significant adaptation in angiogenic signaling.

Impact of integrated IGF, VEGF & VEGFR1 alteration

The coordinated changes across all factors suggest a complex interaction among them. The consistently increased VEGFR1 across all tissue compartments and treatment modalities, paired with minimal VEGF expression, suggests adaptation of the ligand-receptor system to preserve angiogenic homeostasis under metabolic stress in GDM cases [32].

Limitations

We recognize several limitations in this study: a small sample size, a single-center design, a cross-sectional approach, and possible confounding factors related to glycemic control or maternal characteristics.

## Conclusions

Our study demonstrates that GDM induces significant and multifaceted changes in placental morphometry, histopathology, and growth factor expression. Marked upregulation of IGF expression and strong VEGFR1 expression indicate altered angiogenic and growth signaling pathways. Strong correlations between morphometric parameters and growth factor expression suggest coordinated adaptive responses to maintain fetal nutrition despite maternal metabolic dysfunction, though potentially at the cost of long-term placental efficiency. Different GDM treatment modalities produce varying degrees of placental pathology, with insulin-treated cases showing the most marked changes. The findings offer insight into placental molecular adaptations in GDM and may enhance understanding of the mechanisms underlying altered fetal growth. This analysis also reveals that the expression of angiogenic factors in GDM represents a complex, treatment-dependent phenomenon rather than a uniform pathological response. Further research is needed to establish the clinical utility of these findings and develop targeted interventions to optimize placental function in GDM pregnancies. 
